# Validation of a new simple scoring system to predict spontaneous bacterial peritonitis in patients with cirrhosis and ascites

**DOI:** 10.1186/s12876-023-02919-9

**Published:** 2023-08-09

**Authors:** Ngoc Cao Huynh, Thong Duy Vo

**Affiliations:** 1 Department of Gastroenterology, Cho Ray Hospital, 217 Hong Bang, Ward 11, Dis. 5, Ho Chi Minh, 72714 Vietnam; 2 Department of Internal Medicine, University of Medicine and Pharmacy at Ho Chi Minh City, Ho Chi Minh, Vietnam

**Keywords:** Spontaneous bacterial peritonitis, Ascites, Cirrhosis, Predictors, Mansoura scoring system

## Abstract

**Background:**

Recently, a simple scoring system named the Mansoura scoring system was developed to predict spontaneous bacterial peritonitis (SBP) in patients with cirrhosis and ascites. However, the efficacy of this newly developed system has not been extensively investigated. We aimed to validate a new simple scoring system for the rapid diagnosis or exclusion of SBP without paracentesis.

**Methods:**

Adult patients with cirrhosis and ascites admitted to Cho Ray Hospital between November 2021 and May 2022 were included. The area under the receiver operating characteristic (AUROC) curve of the Mansoura simple scoring system for predicting SBP was calculated using the Stata software. Other independent laboratory tests for predicting SBP (C-reactive protein [CRP], neutrophil-to-lymphocyte ratio [NLR], and mean platelet volume [MPV]) were assessed and compared using the Mansoura scoring system.

**Results:**

A total of 121 patients were included in this study. The Mansoura scoring system showed good performance in predicting SBP in patients with cirrhosis and ascites (AUROC:0.89). At the cut-off ≥ 4 points, the scoring system achieved a specificity of 97.7% with a positive predictive value for the diagnosis of SBP of 93.5%. Multivariate analysis was performed using our data and showed that NLR, CRP level, and MPV were independent factors related to SBP.

**Conclusion:**

The Mansoura scoring system demonstrated good performance in predicting SBP in patients with cirrhosis and ascites and may help guide management decisions.

## Background

Spontaneous bacterial peritonitis (SBP) is a bacterial infection of ascitic units without any intra-abdominal, surgically treatable source of infection [1]. SBP is the most frequent bacterial infection in patients with cirrhosis, followed by urinary tract infections, pneumonia, skin and soft tissue infections, and spontaneous bacteremia [2]. When first described, mortality associated with SBP exceeded 90%, but in-hospital mortality was reduced to approximately 20% with early diagnosis and prompt treatment [1, 3]. Society guidelines recommend that a diagnostic paracentesis should be performed as soon as a patient with cirrhosis and ascites is hospitalized urgently for any reason, even in the absence of symptoms suggestive of infection [1, 3, 4]. A delayed diagnosis increases in-hospital mortality; with each hour of delay in paracentesis to diagnose SBP, the mortality increased by 3.3% [5]. However, a study in the United States showed that the rate of paracentesis in clinical practice is still suboptimal; only 66% of patients with cirrhosis and ascites undergo paracentesis in the first 24 h after admission [6]. Most patients who do not undergo paracentesis are in the following categories: elderly, many comorbidities, weekend hospitalizations, hospitalizations at private health facilities, and those with contraindications to paracentesis.

Routine paracentesis cannot be performed in all patients, and delaying diagnosis increases mortality. Therefore, it is necessary to find a non-invasive, highly accurate tool for the diagnosis of SBP. Clinical risk factors for SBP development, such as history of SBP [7], variceal hemorrhage [8], and use of proton pump inhibitors [9] are well known. Numerous laboratory tests have been proposed as SBP predictors, including C-reactive protein [CRP] [10–12], neutrophil-to-lymphocyte ratio [NLR], mean platelet volume [MPV] [12, 13], platelet count [11, 14], serum creatinine level [15] but the data is not consistent. Wehmeyer et al. [11] and Piotrowski et al. [16] proposed a model to predict SBP by combining clinical and laboratory parameters. Preliminary results show the usefulness of the scoring system in predicting SBP.

Recently, the authors at the University of Mansoura retrospectively investigated patients with cirrhosis and ascites and developed the Mansoura scoring system for early diagnosis of SBP without waiting for peritoneal fluid analysis results [17]. The Mansoura scoring system is calculated as a weighted sum of four categories (age, MPV, NLR with one point each and CRP with two points), yielding 0–5 points (Table 1). The scoring system achieved a specificity of 98.2% with a positive predictive value for the diagnosis of SBP of 88.1% (score ≥ 4). At a threshold of one point, the negative predictive value was 97.5%, with a sensitivity of 92.9%.

**Table 1:** Mansoura scoring system

Parameter	“Cut-off”	Scoring points
Age	≥ 55 years	1
MPV	≥ 8.5 fL	1
NLR	≥ 2.5	1
CRP	≥ 40 mg/L	2

MPV, mean platelet volume; NLR, neutrophil-to-lymphocyte ratio; CRP, C-reactive protein

t

It is unclear whether the Mansoura scoring system functions properly in cohorts of patients with different medical, racial, climatic, or geographical backgrounds. In Vietnam, cirrhosis is mainly caused by alcohol and the hepatitis B virus. Thus, in this study, we aimed to validate the Mansoura scoring system in patients presenting with cirrhosis and ascites at the Cho Ray Hospital. Our goal was to define the accuracy of SBP prediction.

## Methods

### Study design

This prospective study included all adult patients with cirrhosis and ascites admitted to the Department of Gastroenterology at Cho Ray Hospital between November 2021 and May 2022. This study was approved by the Ethics Committee of the University of Medicine and Pharmacy at Ho Chi Minh City. All patients who provided informed consent were included in this study.

### Study population

The inclusion criteria were as follows: (a) patients with cirrhosis, (b) presence of ascites, and (c) age > 18 years. The exclusion criteria were as follows: (a) use of antibiotics in the previous two weeks or prophylaxis for SBP before admission; (b) ascites without portal hypertension, such as peritoneal tuberculosis, peritoneal carcinomatosis, congestive heart failure, renal diseases, and pancreatitis, or hemorrhage into ascites; (c) secondary peritonitis; and (d) infections other than SBP, such as pneumonia, urinary tract infection, and skin infection; (e) patients with malignancy; (f) patients with hematologic disease; (g) patient is taking antiplatelet drugs, non-steroidal anti-inflammatory drugs; (h) patients received a platelet or blood transfusion before admission; and (i) patients with diseases associated with increased MPV, for example, diabetes mellitus, cardiovascular disease, hyperthyroidism, immune thrombocytopenia, and myeloproliferative disease.

Within 24 h of admission, the patients underwent paracentesis and were sent for biochemical tests and cell counts. In cases of iatrogenic hemorrhagic ascites (post-paracentesis), the neutrophil counts were.

corrected. One neutrophil was subtracted from the absolute neutrophil count for every 250 red blood cells to yield the “corrected neutrophil count”. The diagnosis of SBP is based on neutrophil count in ascitic fluid of > 250/mm^3^, regardless of the results of ascitic culture [1]. Data were collected for each patient through their medical history, clinical examination, and laboratory results. Based on these data, the Mansoura score was calculated upon admission (Table 1). The Child-Pugh classification and Model for End-stage Liver Disease score system were calculated using an established formula to determine the severity of hepatic decompensation [18, 19].

All patients included in the study had cirrhosis and ascites. Therefore, all the patients had decompensated cirrhosis. Cirrhosis can be diagnosed based on the clinical indicators of severe liver disease, imaging techniques, endoscopic findings, and biochemical indicators of portal hypertension.

### Statistical analysis

We calculated the Mansoura scoring system and analyzed its predictive performance of the scoring system at the optimal cut-off value using the area under the receiver operating characteristic (AUROC) curve. Univariate and multivariate logistic regression analyses were performed to evaluate the risk factors for predicting SBP in patients with cirrhosis and ascites. Statistical significance was set at p < 0.05. All analyses were performed using standard statistical software Stata version 15.1 (Stata Corp LP, College Station, Texas, USA.

## Results

### Patient characteristics

A total of 121 patients were included; 86 (71.1%) were men and 35 (28.9%) were women with a mean age of 57.1 ± 13.6 years. Thirty-four of 121 patients (28.1%) were diagnosed with SBP.

The detailed characteristics of all included patients are shown in Table 2.

**Table 2:** Baseline characteristics of the studied groups

Variable	SBP present *(n = 34) Mean ± SD, or N(%)*	SBP absent *(n = 87) Mean ± SD, or N(%)*	*p-*value
Sex (male/female)	29/5	57/30	0.03
Age	55.4 ± 15.0	57.8 ± 13.1	0.39
**Etiology of Cirrhosis**			
Alcohol	12 (35.3%)	34 (39.0%)	0.86
Hepatitis B virus	13 (38.2%)	30 (34.5%)	
Hepatitis C virus	2 (5.9%)	6 (6.9%)	
Other	7 (20.6%)	17 (19.6%)	
Child – Pugh score			
Child – Pugh B	3 (9.9%)	35.6%)	0.03
Child – Pugh C	31 (91.1%)	56 (64.4%)	
MELD score	25.7 ± 10.6	19.7 ± 9.6	0.002
Fever	17 (50%)	0 (0%)	< 0.001
Abdominal pain	21 (61.7%)	17 (19.6%)	< 0.001
Abdominal tenderness	17 (50%)	2 (2.3%)	< 0.001
Hepatic encephalopathy	5 (14.7%)	9 (10.3%)	0.5
Diarrhea	7 (20.6%)	5 (5.8%)	0.01
Nausea/vomiting	2 (5.9%)	1 (1.2%)	0.19
WBCs (103/mm3)	12.0 ± 6.3	7.3 ± 4.0	0.46
NLR	18.0 ± 16.5	7.4 ± 15.7	0.04
MPV (fL)	10.2 ± 1.1	9.7 ± 1.2	0.02
Platelet Count [10^3^/µL]	109.5 ± 83.6	112.8 ± 61.8	0.8
INR	2.3 ± 1.3	1.8 ± 0.9	0.07
CRP (mg/L)	90.7 ± 56.7	18.7 ± 11.9	< 0.001
Bilirubin (mg/dL)	12.2 ± 11.1	8.0 ± 9.4	0.48
Albumin (g/dL)	2.3 ± 0.3	2.5 ± 0.5	0.07
Creatinine (mg/dL)	1.6 ± 1.2	1.1 ± 1.0	0.64
AST (U/L)	106.8 ± 101.7	133.4 ± 196.3	0.3
ALT (U/L)	76.9 ± 105.7	98.3 ± 239.8	0.4

ALT, alanine aminotransferase; AST, aspartate aminotransferase; CRP, C-reactive protein; INR, international normalized ratio; MELD, Model for End-stage Liver Disease; MPV, mean platelet volume; NLR, neutrophil-to-lymphocyte ratio; SBP, spontaneous bacterial peritonitis; WBC, white blood cell

### Multivariate analysis for prediction of spontaneous bacterial peritonitis

**Fig. 1:**
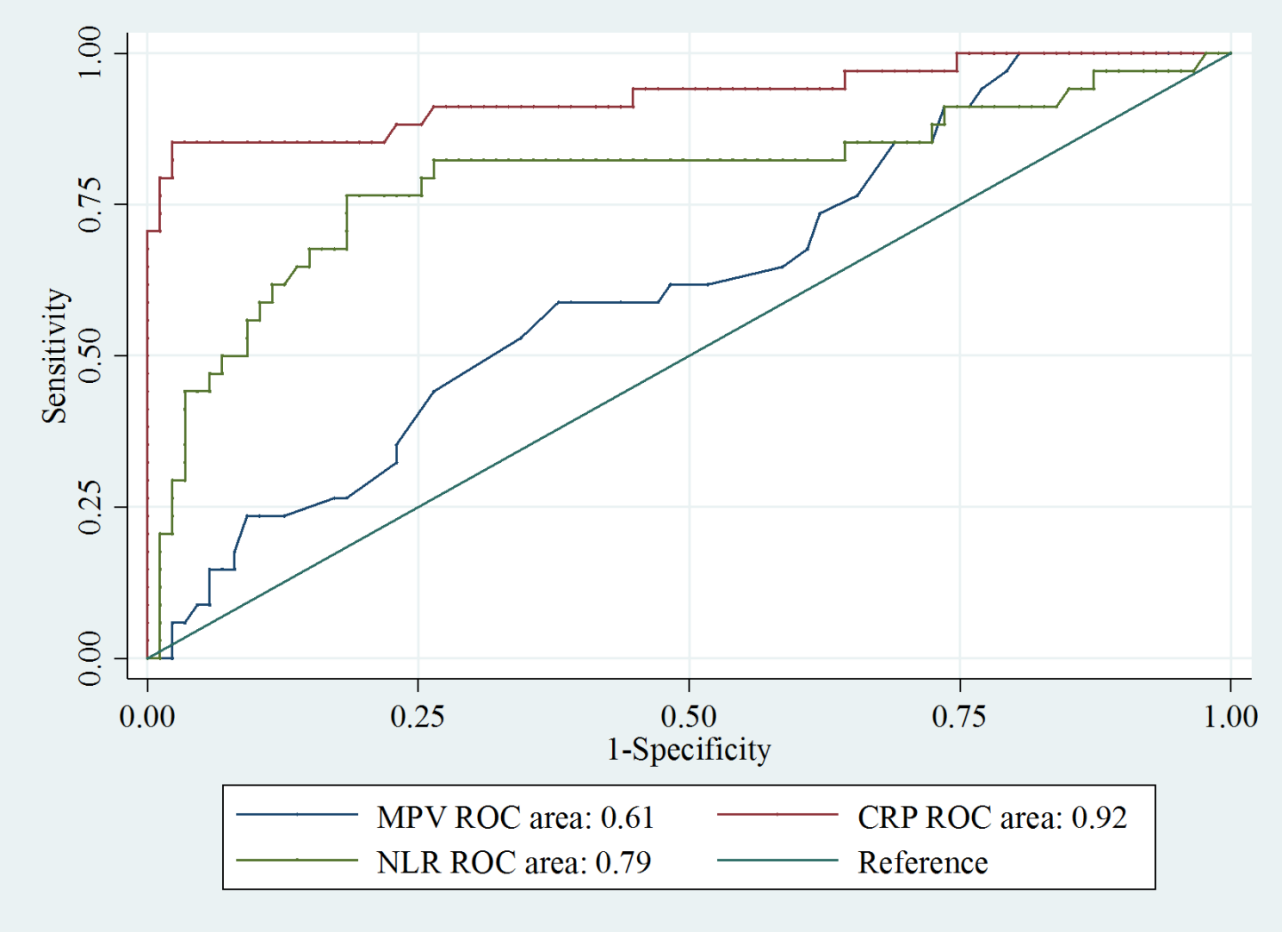
Comparison of NLR, MPV, and CRP performance in the prediction of SBP in patients with cirrhosis and ascites

In analyzing univariate, white blood cells, NLR, MPV, CRP, total Bilirubin, serum albumin, serum creatinine, MELD score, and Child-Pugh score are factors related to SBP. However, in multivariate analysis, we found that NLR, MPV, and CRP were independent variables associated with SBP (Table 2). The performances of the variables are compared and summarized in Fig. 1.

The ROC curve for the specificity and sensitivity of serum CRP showed that at a cut-off value of at least 42.4 mg/L, CRP had 98% specificity and 85% sensitivity for diagnosing SBP (AUC = 0.92; *p* < 0.001).

The ROC curve for the specificity and sensitivity of MPV showed that at a cut-off value of at least 10.1 fL, MPV had 62% specificity and 59% sensitivity for diagnosing SBP (AUC = 0.61; *p* = 0.02).

The ROC curve for the specificity and sensitivity of NLR showed that at a cut-off value of at least 9.2, NLR had 82% specificity and 76% sensitivity for detecting SBP (AUC = 0.79; *p* = 0.04).

The same technique (ROC curves) was used to establish potential thresholds for white blood cell count, platelet count, international normalized ratio, albumin, bilirubin, creatinine, and liver enzymes; however, they did not distinguish between controls and SBP participants (data not shown).

### Diagnostic accuracy of the Mansoura scoring system

Among patients with cirrhosis and ascites, the Mansoura scoring system at different cut-off points showed the ability to diagnose and rule out SBP.

At a cut-off of 1 scoring point, the PPV and the NPV were 28.6% and 100%, respectively. As a result, an SBP was accurately excluded in 100% of the patients with a score of 0 points. In this study, false-negative test results were observed in two of the 12 participants with SBP at a cut-off of 1 point.

At a cut-off of 3 points, the NPV was 92.6% with a PPV of 44.8%, and at a cut-off of 4 points, the NPV was 94.4% with a PPV of 93.5%; all participants with a score of 5 complained of SBP (PPV 100% at a cut-off of 5).

**Fig. 2:**
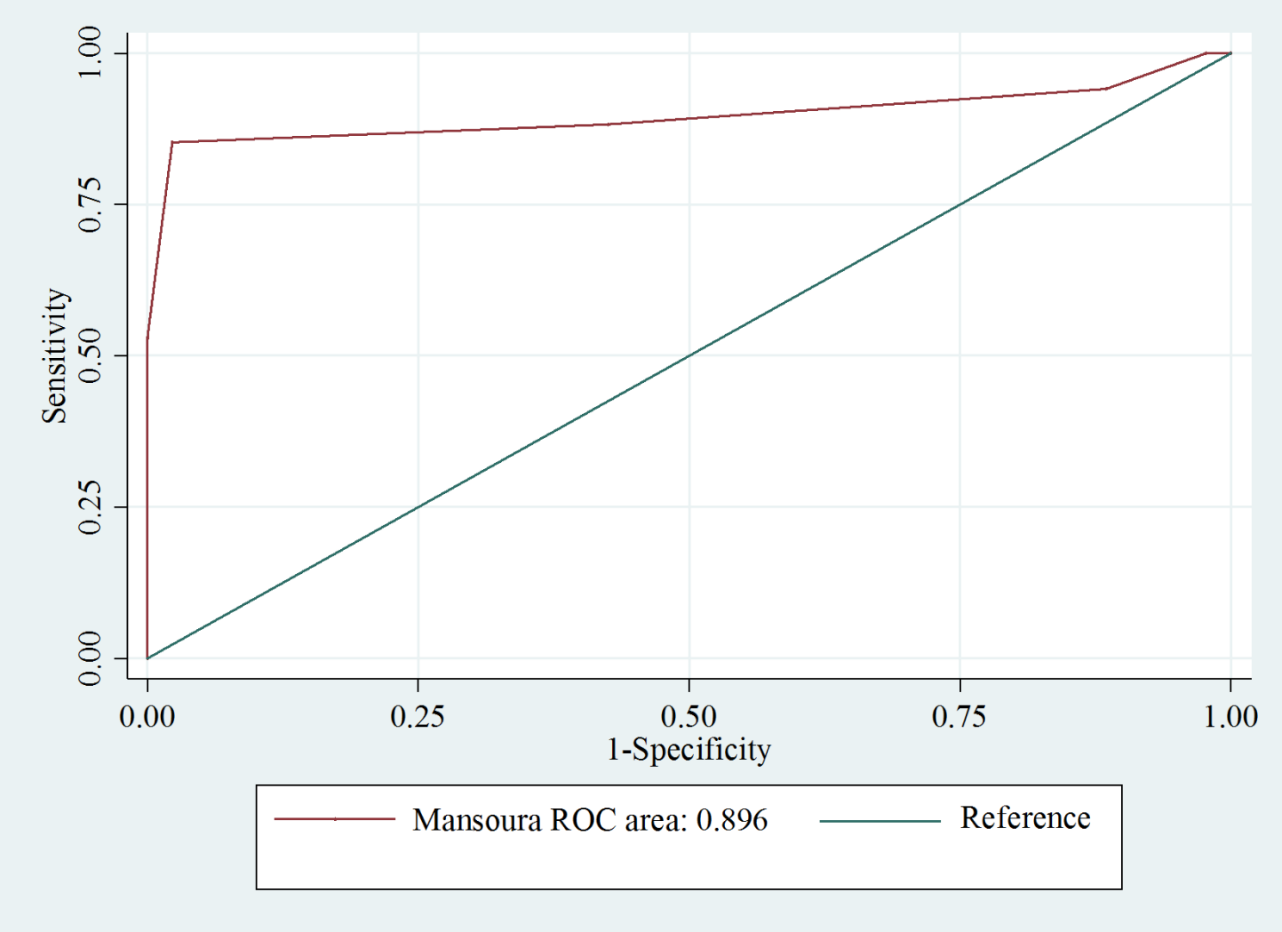
Diagnostic performance of the Mansoura scoring system

The AUC of the Mansoura scoring system was 0.89, with a sensitivity of 85.3%, specificity of 97.7%, PPV of 93.5%, and NPV of 94.4%, at an optimal cut-off score of 4. Patients were classified into two risk groups according to the optimal cut-off value: high probability (score, 4–5) and low probability (score, 0–3). Of the 32 patients with a Mansoura score ≥ 4, 29 (93.5%) had SBP. Table 3 presents the results of the study. Figure 2 shows the diagnostic performance of the Mansoura scoring system.

**Table 3:** Diagnostic validity of the scoring system at different cut-offs

Cut-off	Sensitivity (95% CI) (%)	Specificity (95% CI) (%)	PPV (95% CI) (%)	NPV (95% CI) (%)
1	100 (89.7– 100)	2.3 (0.28–8.0)	28.6 (20.7–37.6)	100 (15.8–100)
2	94.1 (80.3–99,3)	11.5 (5.6–20.1)	29.4 (21.0–38.3)	83.3 (51.6–97.9)
3	88.2 (72.5–96,7)	57.5 (46.4–68.0)	44.8 (32.6–57.4)	92.6 (82.1–97.9)
4	85.3 (68.9–95,0)	97.7 (91.9–99.7)	93.5 (78.6–99.2)	94.4 (87.5–98.2)
5	52.9 (35.1–70,2)	100 (95.8–100)	100 (81.5–100)	84.5 (76.0–90.9)

95% CI, 95% confidence interval; NPV, negative predictive value; PPV, positive predictive value

## Discussion

SBP is a life-threatening condition for which diagnosis and treatment should not be delayed. The Mansoura simple scoring system only uses variables that are collected as part of everyday clinical practice when patients with cirrhosis and ascites are admitted to the hospital and provides cut-offs that are simple to understand and remember [17]. In this study, we evaluated whether this scoring system was effective in predicting SBP in Vietnamese patients with cirrhosis and ascites.

The scoring system was based on a combination of four factors. NLR, CRP, and MPV are markers of inflammation, and advanced age is associated with impaired immune function in the body.

The NLR, which reflects systemic inflammatory reactions, is a crucial marker of homeostasis of the immunological and inflammatory systems. It is a non-invasive marker that can predict the occurrence of nosocomial infections in patients with decompensated cirrhosis [20]. A study by author Piotrowski D shows that the combination of NLR (cut-off point > 2.4) and erythrocyte sedimentation rate has a high accuracy in diagnosing SBP [16]. The combination of NLR and CRP has been demonstrated by Mousa et al. as a simple and non-invasive test for the diagnosis of SBP [10].

MPV has been shown to be a reliable indication of platelet function and thrombopoiesis [21]. According to earlier studies conducted in cirrhotic individuals, MPV may be a predictor of systemic inflammatory responses in SBP [13], [12]. In this study, we found that an MPV cut-off of 10.1 fL had the best predictive value, with a sensitivity, specificity, and AUC of 59%, 62%, and 0.61, respectively.

CRP is a biomarker of systemic inflammatory response and is synthesized during the acute inflammatory phase. Patients with cirrhosis typically have higher basal CRP levels than those without cirrhosis [22]. Various studies have suggested that the cut-off CRP level for the diagnosis of infection in patients with cirrhosis is between 20 and 80 mg/L [10, 22]. In a study by Wehmeyer et al., CRP > 60 mg/L, platelet count > 100 g/L, and age > 60 years were independent variables in predicting SBP [11]. MPV, NLR, and serum CRP levels are the three indicators that can be used to diagnose SBP in cirrhosis and ascites [10, 12, 17]. However, care must be taken when interpreting the CRP results in patients with cirrhosis [22]. In this study, a CRP cut-off point of 42.4 mg/L showed good ability to predict SBP with a sensitivity, specificity, and AUC of 85%, 98%, and 0.91, respectively.

The present study demonstrated that the Mansoura scoring system has excellent performance in predicting SBP in patients with cirrhosis and ascites. The Mansoura scoring system accurately identified a subset of participants with SBP and excluded those with SBP from other groups. Patients with a score of 0 had a very low likelihood of developing SBP (negative predictive value of 100% at a threshold of one scoring point in the study). Higher scores correlated with an increase in SBP diagnosis. At a cut-off of 4 or more points, the positive predictive value was 93.5% (95% confidence interval [CI] 78.6–99.2). The high positive and negative predictive values at this cut-off suggest that patients scoring at least 4 points have SBP. Only two of the 31 patients without SBP received 4 or 5 points.

The results of our study are similar to those of the original study by Abdel-Razik et al. [17]. In both studies, the optimal cut-off of the Mansoura scoring system was 4, and both showed excellent predictive ability for SBP (AUC = 0.891 in the Abdel-Razik et al. study and AUC = 0.896 in this study) (Table 4).

**Table 4:** Comparison between the original study by Abdel-Razik et al. and the current study

	Optimal cut-off	Sensitivity (%)	Specificity (%)	PPV (%)	NPV (%)	AUROC
Abdel-Razik et al. [17]	≥ 4	60.2	98.2	91.9	88.1	0.891
Current study	≥ 4	85.3	97.7	93.5	94.4	0.896

NPV, negative predictive value; PPV, positive predictive value; AUROC, area under the receiver operating characteristic

Our study shows that this new scoring system has many advantages. Firstly, the Mansoura scoring system shows a good ability to diagnose SBP (AUC = 0.89) and was superior to other scoring systems in predicting SBP. Wehmeyer’s scoring system for the diagnosis of SBP has AUC = 0.71 [11] and the combined model with two variables of author Piotrowski has AUC = 0.75 [16]. Secondly, all the variables in the scoring system are readily available, inexpensive, and all show the ability to predict SBP independently. Clinicians can apply this scoring system at the bedside and during the first exam. Thirdly, we classified the patients into the high-risk and low-risk groups based on the Mansoura scoring system cut-off ≥ 4 and ≤ 1. The cut-off value was the same as that published in the original research by author by Abdel-Razik [17].

In Vietnam, at primary medical facilities, paracentesis is not routinely applied. Therefore the scoring system would be helpful for the doctors who examine the patient initially to identify patients who are at high-risk for SBP, allowing for targeted management that could improve outcomes.

### Limitations

Our study has some limitations. First, this was a single-center study, and the number of patients was small. Therefore, a multicenter study with a larger sample size is needed to validate the use of this new scoring system in patients with cirrhosis and ascites. Second, the study included a highly homogeneous population. Third, the scoring system failed to diagnose or rule out SBP in participants who received one or two points. In this study, four patients with 1 or 2 points had SBP that could not be diagnosed by the scoring system.

Although this study had some limitations, we believe that the Mansoura scoring system is a useful tool. The parameters included in this score are simple to assess in the daily examination, and it is possible to quickly diagnose or rule out SBP in many patients with sufficient accuracy. This scoring system is suitable for physicians who treat patients with cirrhosis and ascites to quickly stratify the risk of SBP and to have an appropriate management attitude, aiding physicians in determining which patients require immediate antibiotic therapy, especially when a quick and safe paracentesis is not available and there is a lack of experience with this procedure.

## Conclusion

In this validation study, we found that the Mansoura scoring system performed well in predicting SBP in patients with cirrhosis and ascites. Thus, the Mansoura scoring system shows promise as a first-line modality for the initial diagnosis of SBP under initial examination conditions, especially if prompt paracentesis is not available or cannot be performed safely (e.g., at a private practice or due to lack of experience in this technique).

## Data Availability

The datasets used and/or analysed during the current study are available from the corresponding author on reasonable request.

## Abbreviations

IQR: Interquartile range
SD: Standard deviation
OR: Odds ratio
CI: Confidence Interval
